# Influence of interferon-based drugs on immunological indices in specific prevention

**DOI:** 10.14202/vetworld.2020.238-244

**Published:** 2020-02-10

**Authors:** Alfia Andreeva, Oksana Nikolaeva, Oleg Altynbekov, Chulpan Galieva, Kseniia Ilina

**Affiliations:** Department of Infectious Diseases, Zoohygiene and Veterinary Sanitary Inspection of Federal State Budget Educational Institution of Higher Education “Bashkir State Agrarian University”, Ufa 450001, Republic Bashkortostan, Russian Federation

**Keywords:** antibody titer, coronavirus, immunoglobulins A, M, and G, interferon, *Rotavirus*, vaccinations

## Abstract

**Aim:**

The research aimed to study the effect of interferon (IFN)-based drugs on the behavior of immunological parameters in calves during the specific prevention of associative infections.

**Materials and Methods:**

The object of research was 45 black motley cows and their calves from birth to 2 months of life. Serum and colostrum samples were screened for antibodies against *Rotavirus*, diarrhea, and coronavirus using serological methods. The testing was performed before vaccination, 40 days before calving, 20 days before calving, and before calving. Colostrum samples were taken during the first milk yield. Serum samples from calves were drawn before colostrum feeding as well as at 7, 14, and 21 days, and 1 and 2 months of age. To measure the level of immunoglobulins A, M, and G, additional serum samples were collected from calves at 25, 35, 65, and 75 days after birth.

**Results:**

Giving pregnant cows, an IFN-based drug at a dose of 1 ml/kg 48 h before vaccination results in the development and accumulation of antibodies to *Rotavirus*, coronavirus, and viral diarrhea (VD) in the colostrum, with a titer of 7.6±0.3 log_2_, 5.8±0.34 log_2_, and 4.4±0.18 log_2_, respectively. It indicates an increase in the antigenic activity of the multivalent vaccine.

**Conclusion:**

IFN-based drugs enhance the protective effect of vaccination against associative infections in the newborn calves. They stimulate a rise in the titer of antibodies to *Rotavirus*, coronavirus, VD, and mucosal disease complex as well as an increase in immunoglobulins A, M, and G.

## Introduction

The most effective protection against infectious diseases involves specific prevention, i.e. vaccination. However, it could be sometimes ineffective. This problem can be solved using immune-stimulating drugs. Immune-stimulants cause changes in the activity of humoral and cellular components of the immune system, thereby strengthening the immune response [1-3]. To this end, a promising group of immune-stimulating agents includes those based on natural interferons (IFNs) [4,5].

In terms of veterinary medicine, IFNs are of interest due to their antiviral and anti-inflammatory properties. IFNs can claim a role in the therapeutic and preventive treatment of viral, bacterial, and mixed bacterial-viral infections. Moreover, they are highly effective immune-modulating and anti-stress agents. They can modify the action of other treatment medications, for example, antibiotics, tens of times amplifying their antibacterial effect, and neutralizing the negative impact on the immune system [2,6].

The successful production of IFN-inducing strains, sophisticated methods to refold and purify proteins, and new and unique formulations has resulted in a wide range of mono- and multicomponent veterinary preparations to address different issues in veterinary science. The main distinguishing feature of all developed drugs is their species specificity, corresponding to the species specificity of the IFNs included in their composition. These are unique preparations. Their active principle of protective proteins is completely identical to the animal’s ones. Thus, animals can be treated not with foreign substances, but with their protective agents, simply increasing their concentration in the body by inducing IFN-based preparations at the right time [4,7].

In total, there are three groups of veterinary drugs:

i. Monocomponent preparations with IFN as the active ingredient
ii. Bicomponent preparations with IFN and an antibacterial agent (antibiotic) as the active substance to treat mixed bacterial-viral infections
iii. Multicomponent preparations with IFN and a complex of Vitamins A, D_3_, E, and C as the active ingredient in their optimum physiological ratios [7-10].

The antiviral effect of IFN appears inside the cell and is associated with suppression of virus reproduction. IFN does not act on extracellular viruses and their adsorption [11-13]. IFN is not active inside the cell where it is produced. It must be secreted and then reabsorbed by the cells, where it binds to a corresponding receptor on the cell membrane. There are different types of protein receptors. After binding to cell receptors, the complexes of IFNs diffuse and aggregate on the cell surface. Then, they undergo endocytosis, which occurs after 1-2 h. Getting inside the cells, they activate processes that result in the antiviral state and determine different effects on metabolism. Namely, there takes place the production of different intracellular proteins with oligoadenylate synthetase and protein kinase being the most important and well-studied. These enzyme systems ultimately induce the spread (reproduction) of the virus *in vivo*, inactivating early viral mRNAs. It inhibits the synthesis of viral proteins on cell ribosomes during viral nucleic acid replication [14,15].

IFN or an inducer of endogenous IFN activates the absorption of monocytes and tissue macrophages. A higher ingestion rate is related to increased activity of macrophage elements. It is not accompanied by growth in the number of phagocytic cells [9,13]. Disease prevention and treatment with the help of IFNs are developed in two directions: The introduction of ready-made forms of IFN (exogenous interferonization) and the stimulation of the IFN production by cells of the body under the influence of inducers (endogenous interferonization) [13].

Based on the preceding, the search and use of IFNs and their inducers with a wide range of antiviral and immune-modulating effects are a promising and necessary direction of immune pharmacology. Meanwhile, there are several main directions to use IFNs having been outlined in veterinary practice: The use of exogenous IFNs, the use of endogenous IFN inducers, and the combined use of IFNs with chemotherapeutic drugs and vaccines.

The research aimed to study the effect of IFN-based drugs on calf immunological parameters in the specific prophylaxis of associative infections.

## Materials and Methods

### Ethical approval

The study was conducted under the ethical principles approved by the Animal Experiments Ethics Committee, Federal State Budgetary Educational Institution of Higher Professional Education “Bashkir State Agrarian University” (Protocol No. 8 of 28.03.2019).

### Study period and location

The research was carried out from 2014 to 2019 on a livestock farm in the Chishminsky District of the Republic of Bashkortostan of the Russian Federation.

### Animals and donors

The object of research was 45 black motley cows and their calves from birth to 2 months of life.The animals for research were selected by the analog approach. They were fed and kept in the same conditions. Animals for research were selected identical in age and live weight; calves up to two months of age received a daily diet; the animals were in a room that meets the zoohygienic requirements for keeping cattle.

The work was based on:

1. Inactivated combined vaccine “Combovac” against infectious rhinotracheitis, parainfluenza-3, viral diarrhea (VD), respiratory syncytial, and *Rotavirus* and coronavirus diseases of calves (Scientific production community “Narvak,” Moscow)
2. “IFN bovine recombinant” (IBR) (Scientific production center BelAgroGen, LLC, Belorussia)
3. “Immunate” (Scientific production center BelAgroGen, LLC, Belorussia).

The mother cows of the first (experimental) group (n=15), 48 h before vaccination, were once injected with an immunostimulant IBR at a dose of 1 ml/kg of body weight. Vaccination was carried out twice according to the instructions: They were injected subcutaneously into the neck at a dose of 2 ml 40 days before calving for the 1^st^ time and 20 days before calving for the 2^nd^ time. Animals of the second (experimental) (n=15) group, the vaccine was administered according to the same scheme. Forty-eight hours before vaccination, an immunomodulator Immunate was injected at a dose of 5 ml per animal. The control (n=15) cows were pregnant vaccinated ones. They were not given immunostimulants.

The newborn calves were divided into nine groups following the division of mother cows in the first scientific experiment, five animals each. The calves from each group of cows were divided into three groups. In the first group of calves obtained from cows of the first (control) group, drugs were not used, which was a control for the second stage of research. Furthermore, immunostimulating drugs were not used in the fourth and seventh (experimental) groups of calves obtained from mother cows of the second and third (experimental) groups. The calves of the second, fifth, and eighth (experimental) groups were used with an IBR immunostimulant at a dose of 1 ml/10 kg of body weight on the 1^st^ day after birth twice with an interval of 48 h. The calves of the third, sixth, and ninth (experimental) groups were injected with the preparation Immunate in a dose of 2 ml per animal.

The vaccination of mother cows with the “Combovac” vaccine was carried out twice according to the instructions. They were injected subcutaneously in the neck area at a dose of 2 ml 40 days before calving for the 1^st^ time and 20 days before calving for the 2^nd^ time. The vaccination of the obtained calves with the “Combovac” vaccine against associative infections was carried out twice. One milliliter of the vaccine was injected into the neck area subcutaneously at the age of 30 days, with an interval of 20 days.

### Blood sampling, isolation, and processing serological tests

Serum and colostrum samples from mother cows were assayed for antibodies to *Rotavirus*, diarrhea, and coronavirus using the serological methods. The testing was carried out before vaccination, 40 days before calving, 20 days before calving, and before calving. Colostrum samples were taken during the first milk yield.

To assess colostral immunity in calves born from vaccinated cows, serum samples were drawn before colostrum feeding as well as at 7, 14, and 21 days, and 1 and 2 months of age. Serum and colostrum samples from control and experimental animals were assayed for antibodies to *Rotavirus* and VD by the enzyme-linked immunosorbent assay, to coronavirus by the hemagglutination inhibition assay. The total amount of immunoglobulins A, M, and G in the blood sera of animals at 25, 35, 65, and 75 days after birth was determined by quantitative methods such as the radial immunodiffusion. The number of immunoglobulins (mg/ml) was determined by a calibration curve reflecting the relationship between the logarithm of the concentration of immunoglobulins in serum and the diameter of the precipitation ring.

### Statistical analysis

The experimental data were processed with the statistical analysis package for Microsoft Excel^®^. The significance of differences between groups was evaluated using Student’s t-test from p≤0.05 to p≤0.001.

## Results

**Figure-1:**
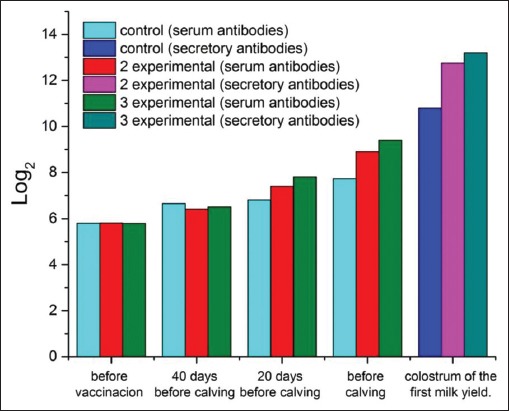
Dynamics of the titer of antibodies to *Rotavirus* in the blood serum of cows and colostrum of the first milk yield.

After vaccination, there was a significant increase in *Rotavirus* antibody titers in the blood serum of pregnant cows if compared to the initial amount (before vaccination) (Figure-1).

The initial titer of antibodies (before vaccination) was 5.8 1og_2_. By the time of calving, it increased in animals of the third group amounted to 9.4±0.34 (p<0.01). In the control group, antibody titers in cows before calving were 7.72±0.25 log_2_. In animals of the second (experimental) group, the increase in post-vaccination antibodies (calving) was 2.6 log_2_ (p<0.01). In the colostrum of the first milk yield in calving cows of the control group, the *Rotavirus* antibody amounted to 10.8±0.31 log_2_. In the second (experimental) group, the same indicator exceeded the control by 1.95 log_2_. In the third (experimental) group, the titers of colostrum antibodies in experimental cows exceeded the control values by 2.4 log_2_ (Figure-1).

**Figure-2:**
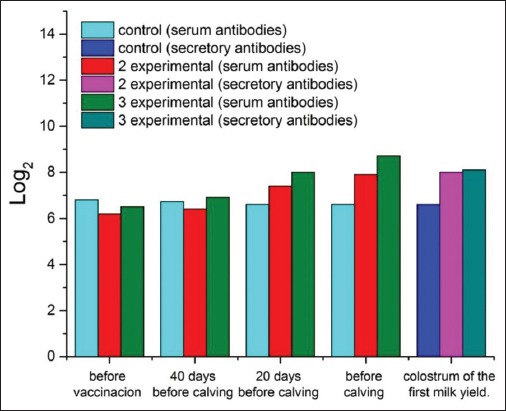
Dynamics of the titer of antibodies to coronavirus in the blood serum of cows and colostrum of the first milk yield.

In the first (control) group, the number of coronavirus antibodies did not change significantly during the experiment. It slightly decreased by the time of calving (Figure-2). In the third (experimental) group, there was the highest increase in post-vaccination titer. It was 2.2 1og_2_ higher than the baseline.

Serum and colostrum samples of the control group (non-immunostimulated cows) proved that coronavirus antibodies were present in all animals before calving (Figure-2). On the 2^nd^ day, the level of antibodies decreased by 2-3 times, and only a small amount of them was found in milk on the 3^rd^ day of lactation in 15-20% of animals. In animals of the second and third groups, the coronavirus antibody titers were 8.0±0.15 and 8.1±0.17 1og_2_ (p<0.01) on the day of calving; 6.2±0.2 and 6.4±0.22, 4.6±0.24 and 4.7±0.21, and 1.8±0.31 and 1.85±0.29 1og_2_ (p<0.01) on 2, 3, and 4 days of lactation, respectively. By the 5^th^-7^th^ day, antibodies to the indicated antigens were detected in titers of 0.8±0.13. Further, the titer of coronavirus antibodies gradually decreases, and by the 8^th^ day of lactation, they are not detected.

**Figure-3:**
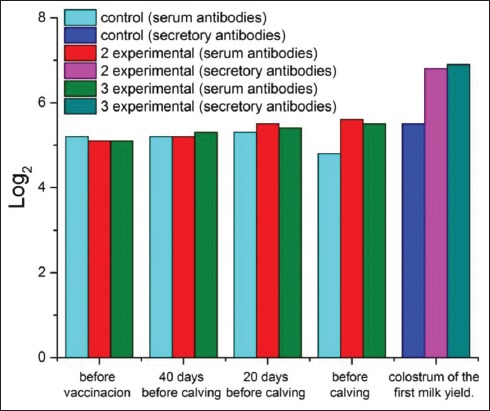
Dynamics of the titer of antibodies to viral diarrhea in the blood serum of cows and colostrum of the first milk yield.

Before vaccination, the titer of antibodies to VD in all groups of cows ranged from 5.1 to 5.2 log_2_ (Figure-3). In the first (control) group, this indicator did not change significantly after the first and second vaccinations. It decreased by the time of calving. In the group of animals that used the IBR immunostimulant (second group), the average titer of VD antibodies before calving exceeded the pre-vaccination antibody titers by 0.5 1og_2_. In the control group, the average titer of VD antibodies before calving exceeded the pre-vaccination titer by 0.8 1og_2_. In the third group, the titers of humoral antibodies after immunization with the Immunate increased by 0.4 1og_2_. The difference in antibody titers before calving between the control and the third group was 0.7 1og_2_ (p<0.05).

The titers of colostrum secretory antibodies to VD in the second (experimental) group exceeded the control values by 1.3 log_2_. In the third (experimental) group, they surpassed the control values by 1.4 log_2_.

Before colostrum intake, no calves had *Rotavirus* antibodies in their blood serum (Table-1).

**Table-1:** The dynamics of humoral antibodies in calves to *Rotavirus*.

Animal group	Titers of antibodies in blood serum, 1og_2_±m					

	1^st^ day	7^th^ day	14^th^ day	21^st^ day	30^th^ day	60^th^ day
First (control)	4.3±0.25	6.3±0.28	4.8±0.17	4.2±0.33	4.13±0.19	3.9±0.22
Second (experimental)	5.1±0.28	6.4±0.39	5.8±0.21	5.2±0.38	4.8±0.27	4.6±0.29
Third (experimental)	5.03±0.31	6.4±0.25	5.6±0.42	5.05±0.16	4.7±0.31	4.4±0.33
Fourth (experimental)	6.0±0.29*	7.1±0.22*	6.6±0.31*	5.7±0.29*	5.5±0.19*	5.1±0.18*
Fifth (experimental)	6.7±0.21*	7.8±0.33*	7.5±0.36*	6.8±0.28*	6.4±0.21*	6.0±0.24*
Sixth (experimental)	8.1±0.33**	8.9±0.31**	8.01±0.23**	7.9±0.21**	7.8±0.27**	7.6±0.3**
Seventh (experimental)	5.8±0.41*	6.83±0.39*	6.6±0.28*	5.3±0.28*	5.1±0.29*	4.9±0.24*
Eighth (experimental)	7.01±0.25**	8.2±0.27**	7.6±0.24**	7.45±0.26**	7.2±0.33**	7.1±0.35**
Ninth (experimental)	7.2±0.22**	8.6±0.3**	7.83±0.29**	7.6±0.34**	7.54±0.41**	7.35±0.21**

Level of reliability

* p<0.05;

** p<0.01

As one can see, by the end of the 1^st^ week after birth, *Rotavirus* antibodies reached their maximum values. A month after birth, the level of these antibodies slightly decreased in all groups of calves. During the 2^nd^ month after birth, the titer of humoral antibodies increased in the experimental group and decreased in the control one. Typically, the values of *Rotavirus*-specific antibody titers in the experimental group significantly exceeded those in the control group. The maximum difference in this indicator between the experimental and control group was recorded for calves in the sixth and ninth experimental groups.

Before colostrum intake, no calves had coronavirus antibodies in their blood serum (Table-2).

**Table-2:** The dynamics of humoral antibodies in calves to coronavirus.

Animal group	Titers of antibodies in blood serum, 1og_2_±m					

	1^st^ day	7^th^ day	14^th^ day	21^st^ day	30^th^ day	60^th^ day
First (control)	4.6±0.12	6.04±0.38	6.0±0.19	5.9±0.3	4.75±0.26	3.4±0.41
Second (experimental)	5.1±0.33	8.0±0.19*	7.9±0.23	7.85±0.31	6.6±0.29	5.3±0.28
Third (experimental)	5.3±0.27	8.35±0.26*	8.2±0.39	8.1±0.19	7.5±0.35	5.4±0.31
Fourth (experimental)	6.1±0.24	9.1±0.19*	9.0±0.26*	8.95±0.21*	7.7±0.23*	5.0±0.24*
Fifth (experimental)	6.3±0.18	9.3±0.31*	9.2±0.29*	9.15±0.28*	7.9±0.13*	5.6±0.27*
Sixth (experimental)	6.4±0.31	9.8±0.27**	9.7±0.21**	9.5±0.38**	8.1±0.32**	5.8±0.34**
Seventh (experimental)	6.2±0.28	9.1±0.27*	8.9±0.24*	8.9±0.33*	7.6±0.18*	5.1±0.23*
Eighth (experimental)	6.3±0.36	9.4±0.35**	9.3±0.24**	9.2±0.31**	7.8±0.39**	5.7±0.31**
Ninth (experimental)	6.5±0.32	9.7±0.26**	9.7±0.3**	9.5±0.28**	8.1±0.32**	5.75±0.28**

Level of reliability

* p<0.05;

** p<0.01

The dynamics of serum antibodies in calves was as follows: Antibody titers reached their maximum values by the end of the 1^st^ week of animal life, the highest level was indicated in calves of the sixth, eighth, and ninth groups. Then, the values gradually decreased by the end of the observation period (2 months).

In calves of the control and experimental groups, antibodies to VD were absent before colostrum administration (Table-3).

**Table-3:** The dynamics of humoral antibodies in calves to viral diarrhea.

Animal group	Titers of antibodies in blood serum, log_2_±m					

	1^st^ day	7^th^ day	14^th^ day	21^st^ day	30^th^ day	60^th^ day
First (control)	3.5±0.21	4.05±0.26	3.6±0.27	3.4±0.4	2.8±0.34	1.9±0.22
Second (experimental)	4.2±0.26	5.41±0.19	5.3±0.38	4.9±0.29	4.6±0.35	3.6±0.29
Third (experimental)	4.3±0.38	5.38±0.22	5.2±0.39	5.1±0.26	4.5±0.41	3.7±0.38
Fourth (experimental)	4.2±0.19	5.18±0.21	5.0±0.33	4.8±0.18	4.4±0.21	3.5±0.27
Fifth (experimental)	4.3±0.16	6.1±0.32*	5.5±0.4	5.2±0.36	4.7±0.31	3.8±0.26
Sixth (experimental)	4.4±0.33	6.2±0.28*	6.1±0.29*	5.95±0.4*	5.4±0.33*	4.4±0.18*
Seventh (experimental)	4.2±0.27	5.2±0.31	5.1±0.24	4.8±0.32	4.3±0.16	3.6±0.21
Eighth (experimental)	4.3±0.31	6.3±0.18*	6.1±0.29*	5.9±0.17*	5.3±0.28*	4.2±0.34*
Ninth (experimental)	4.4±0.26	6.4±0.29*	6.2±0.28**	5.9±0.39*	5.3±0.33*	4.3±0.27*

Level of reliability

* p<0.05;

** p<0.01

## Discussion

By the end of the 1^st^ week of life, the titer of humoral antibodies in the experimental groups of calves reached the maximum values. The highest deviations from the values of the control group were recorded in calves obtained from mother cows immunostimulated before vaccination during pregnancy. These calves were also given immunostimulants after birth. In the eighth and ninth (experimental) groups, the indicator had a maximum mark and exceeded the control by 2.25 and 2.35 log_2_, respectively. Subsequently, antibody titers in animals of all groups gradually decreased, but the experimental ones steadily exceeded the control indices.

Danish scholars [16] also found the correlation between IFN and the increase in antibody titer, but their study was devoted to poultry. Some scientists [13,17] conducted studies on farm animals to prevent and treat viral and bacterial infections with the use of IFNs and proved their effect positive.

The dynamics of immunoglobulins was monitored for 75 days (Table-4). For all the periods of the study, the content of immunoglobulins A, M, and G in the blood serum of the calves of the control group was lower than similar indicators of calves of the experimental groups. The maximum difference between these indicators with control was found in animals of the sixth and ninth groups. Hence, on the 65^th^ day of the experiment, in the sixth group, the content of immunoglobulins A, M, and G was higher than the control by 0.14, 0.18, and 1.14 mg/ml; in the ninth, by 0.08, 0.31, and 2.86 mg/ml, respectively. However, on the 75^th^ day of the experiment, a decrease in the number of serum immunoglobulins A, M, and G was observed in the studied groups of calves. The maximum decrease was recorded in the control group. In the sixth and ninth groups of calves, the smallest decrease in immunoglobulins A, M, and G was recorded.

**Table-4:** The dynamics of immunoglobulins in the serum of calves.

Animal group	Days of research									

	Background		25^th^		35^th^		65^th^		75^th^	

	Statistical index									

	M±m	p-value	M±m	p-value	M±m	p-value	M±m	p-value	M±m	p-value
Immunoglobulin A, g/l										
left (control)	0.56±0.013		0.58±0.03		0.67±0.03		0.71±0.01		0.61±0.03	
Second (experimental)	0.45±0.013		0.5±0.017		0.65±0.04		0.75±0.02		0.70±0.03	*
Third (experimental)	0.57±0.008		0.59±0.05		0.69±0.02		0.71±0.02	**	0.64±0.05	***
Fourth (experimental)	0.48±0.012		0.5±0.014		0.61±0.018		0.72±0.011		0.69±0.019	
Fifth (experimental)	0.51±0.009		0.53±0.012		0.64±0.021		0.7±0.016		0.67±0.017	
Sixth (experimental)	0.55±0.01		0.58±0.022		0.71±0.019		0.85±0.02	***	0.83±0.03	***
Seventh (experimental)	0.53±0.014		0.55±0.019		0.62±0.015		0.74±0.021		0.68±0.013	
Eighth (experimental)	0.47±0.015		0.56±0.018		0.67±0.012		0.71±0.028		0.69±0.018	
Ninth (experimental)	0.54±0.009		0.58±0.024		0.73±0.015	***	0.79±0.011		0.74±0.013	
Immunoglobulin M, g/l										
First (control)	1.63±0.035		1.67±0.045		1.73±0.046		1.79±0.016		1.7±0.02	
Second (experimental)	1.57±0.03		1.63±0.05		1.75±0.035		1.82±0.025		1.75±0.03	
Third (experimental)	1.64±0.029		1.68±0.038		1.77±0.045		1.81±0.04		1.74±0.05	
Fourth (experimental)	1.61±0.031		1.67±0.032		1.76±0.038		1.84±0.036		1.76±0.018	
Fifth (experimental)	1.60±0.029		1.69±0.033		1.78±0.04		1.91±0.028		1.85±0.021	
Sixth (experimental)	1.59±0.03		1.77±0.029	**	1.86±0.039	*	1.97±0.04	**	1.92±0.02	***
Seventh (experimental)	1.59±0.028		1.66±0.028		1.74±0.035		1.86±0.041		1.78±0.024	
Eighth (experimental)	1.58±0.031		1.75±0.027	***	1.82±0.031	*	1.92±0.038	**	1.84±0.026	**
Ninth (experimental)	1.62±0.027		1.73±0.024	***	1.91±0.033	**	2.1±0.031	***	2.08±0.029	***
Immunoglobulin G, g/l										
First (control)	13.2±0.12		12.75±0.16		13.4±0.13		13.9±0.11		13.0±0.13	
Second (experimental)	12.8±0.16		13.30±0.16		13.84±0.11		14.84±0.1		14.8±0.3	
Third (experimental)	12.6±0.21		12.25±0.11		13.58±0.17		14.58±0.2		14.4±0.17	
Fourth (experimental)	12.7±0.18		13.2±0.19		13.62±0.18		14.61±0.21		14.54±0.18	
Fifth (experimental)	12.6±0.22		13.63±0.14		13.98±0.22		14.77±0.18		14.68±0.13	*
Sixth (experimental)	13.0±0.17		13.95±0.16	***	14.55±0.28	***	15.04±0.21	***	15.0±0.19	
Seventh (experimental)	13.1±0.19		13.4±0.19		14.21±0.26		14.85±0.23		14.7±0.15	**
Eighth (experimental)	12.8±0.17		13.4±0.16		14.38±0.13		14.9±0.17		14.65±0.12	
Ninth (experimental)	12.8±0.23		13.9±0.15	***	15.25±0.17	**	16.76±0.18	***	16.58±0.22	

Difference is reliable with

* p<0.05;

** p<0.01;

*** p<0.001

## Conclusion

Thus, during the research, it was found that:

- Dosing pregnant cows with an IFN-based drug at 1 ml/kg 48 h before vaccination contributes to the accumulation of *Rotavirus*, coronavirus, and VD antibodies in the colostrum, with a titer of 7.6±0.3 log_2_, 5.8±0.34 log_2_, and 4.4±0.18 log_2_, respectively. It indicates an increase in the antigenic activity of the multivalent vaccine
- IFN-based drugs enhance the protective effect of vaccination against *Rotavirus* and coronavirus enteritis, VD, and mucosal diseases in newborn calves. It is achieved through the increase in the titer of associated antibodies and immunoglobulins A, M, and G.

To sum up, the use of IFN-based drugs before vaccination increases the efficiency of vaccination and enhances colostral immunity, helping improve the survival of young animals.

## Authors’ Contributions

AA designed the study, wrote and revised the manuscript, and managed correspondence. ON applied testing. OA collected the results of this test. CG and KI collected the results of this test and wrote the manuscript. All authors read and approved the final manuscript.
